# Integration of radiogenomic features for early prediction of pathological complete response in patients with triple-negative breast cancer and identification of potential therapeutic targets

**DOI:** 10.1186/s12967-022-03452-1

**Published:** 2022-06-07

**Authors:** Ying Zhang, Chao You, Yuchen Pei, Fan Yang, Daqiang Li, Yi-zhou Jiang, Zhimin Shao

**Affiliations:** 1 Department of Breast Surgery, Fudan University Shanghai Cancer Center, 270 Dongan Road, Xuhui District, Shanghai, People’s Republic of China; 2 Department of Oncology, Shanghai Medical College, Fudan University, Shanghai, People’s Republic of China; 3 Department of Radiology, Fudan University Shanghai Cancer Center, Shanghai, People’s Republic of China; 4 Precision Cancer Medicine Center, Fudan University Shanghai Cancer Center, Shanghai, People’s Republic of China

**Keywords:** Radiomics, Genomics, Neoadjuvant chemotherapy, Triple-negative breast cancer, Pathological complete response

## Abstract

**Background:**

We established a radiogenomic model to predict pathological complete response (pCR) in triple-negative breast cancer (TNBC) and explored the association between high-frequency mutations and drug resistance.

**Methods:**

From April 2018 to September 2019, 112 patients who had received neoadjuvant chemotherapy were included. We randomly split the study population into training and validation sets (2:1 ratio). Contrast-enhanced magnetic resonance imaging scans were obtained at baseline and after two cycles of treatment and were used to extract quantitative radiomic features and to construct two radiomics-only models using a light gradient boosting machine. By incorporating the variant allele frequency features obtained from baseline core tissues, a radiogenomic model was constructed to predict pCR. Additionally, we explored the association between recurrent mutations and drug resistance.

**Results:**

The two radiomics-only models showed similar performance with AUCs of 0.71 and 0.73 (*p* = 0.55). The radiogenomic model had a higher predictive ability than the radiomics-only model in the validation set (*p* = 0.04), with a corresponding AUC of 0.87 (0.73–0.91).

Two highly frequent mutations were selected after comparing the mutation sites of pCR and non-pCR populations. The MED23 mutation p.P394H caused epirubicin resistance in vitro (*p* < 0.01). The expression levels of γ-H2A.X, p-ATM and p-CHK2 in MED23 p.P394H cells were significantly lower than those in wild type cells (*p* < 0.01). In the HR repair system, the GFP positivity rate of MED23 p.P394H cells was higher than that in wild-type cells (*p* < 0.01).

**Conclusions:**

The proposed radiogenomic model has the potential to accurately predict pCR in TNBC patients. Epirubicin resistance after MED23 p.P394H mutation might be affected by HR repair through regulation of the p-ATM-γ-H2A.X-p-CHK2 pathway.

**Supplementary Information:**

The online version contains supplementary material available at 10.1186/s12967-022-03452-1.

## Introduction

Triple-negative breast cancer (TNBC) is characterized by the negative expression of estrogen receptor, progesterone receptor, and human epidermal growth factor 2 [1]. Neoadjuvant chemotherapy (NAC) serves as the main therapy for TNBC patients. Patients who achieve pathological complete response (pCR) have a better prognosis than non-pCR patients [2, 3]. However, less than 40% of TNBC patients achieve pCR after the completion of a standard NAC regimen [4]. Therefore, early response prediction is valuable for clinical decisions regarding whether treatment modification is needed to obtain an improved response.

To date, neither traditional clinicopathological nor radiologic features can accurately predict the NAC response. The accuracy of ultrasonography for pCR is only approximately 70% [5]. The change in the maximum standardized uptake value of PET scans before the first two cycles of NAC has been used to predict treatment response, but the area under the curve (AUC) values were less than 0.70 [6]. MRI serves as the standard tool for the surveillance of breast cancer response, but none of the traditional radiologic methods can predict the treatment response with sufficient accuracy [7]. Thus, early and accurate predictions to NAC response remain challenging.

In recent years, the application of radiomics approaches, which are less invasive, has contributed to both cancer diagnosis [8] and response prediction [9–11]. However, most studies have focused on baseline predictions; data on the predictive ability of radiomic features mid-treatment are still lacking. Radiogenomics links imaging features with the genetic profiles of tumors [12, 13]. The integration of complementary data generated from radiomics and genomics might further improve the performance of predictive models [13]. Studies have also expanded to include multiple genetic markers, which has helped to stratify TNBC patients for more tailored therapy [14, 15].

In this study, we attempted to establish a new model based on the light gradient boosting machine (LightGBM) method [16], which combines both radiomic and genomic data for the early prediction of the response to NAC in TNBC patients. Additionally, we explored the relationships between two potential mutation targets and drug resistance in vitro.

## Methods

### Patients and neoadjuvant treatment

**Fig. 1:**
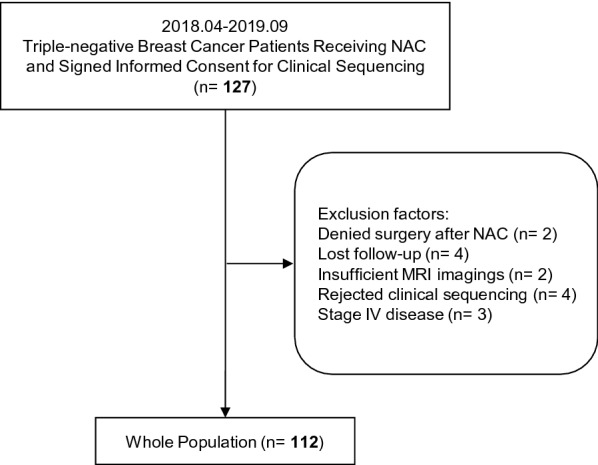
Flow chart

All patients were participants in the previously reported Precision Oncology Program [17] at Fudan University Shanghai Cancer Center and were prospectively recruited. From April 2018 to September 2019, consecutive patients diagnosed with invasive TNBC undergoing NAC who signed an informed consent form for sample collection and clinical sequencing were identified and included in the analysis (Fig. 1). Patients with carcinoma in situ, microinvasive cancer, occult breast cancer, metastatic disease, a prior history of other malignancies and incomplete CE-MRI imaging data were excluded. Patients who had a confirmed TNBC diagnosis through open biopsy and those who were lost to follow-up were ineligible. A triple-negative phenotype was defined as ER/PR negativity (cutoff point: < 1%) and an immunohistochemistry score of 0 or 1 or lack of HER2 amplification by fluorescence in situ hybridization [18].

Four to eight cycles of NAC (taxane-based, anthracycline-based or taxane-and-anthracycline-based regimens) were applied according to NCCN guidelines and clinical trial protocols (NCT02628613 and 04,215,003). The regimens included dose dense (dd) EC-T (epirubicin/cyclophosphamide followed by docetaxel), ddEC-ddP (epirubicin/cyclophosphamide followed by paclitaxel), wPC (paclitaxel/carboplatin), wP (paclitaxel), wPE (paclitaxel/epirubicin) and wNE (vinorelbine/epirubicin). All patients underwent core-needle biopsy and CE-MRI at baseline and were further monitored by CE-MRI every two cycles. PCR was defined as free of invasive cancer burden in either the breast or associated axillary lymph nodes (ypT0/is ypN0).

All tissue samples in this study were obtained after approval by the Fudan University Shanghai Cancer Centre Institutional Review Board (No. 090977–1), and each patient provided written informed consent.

### Radiomic data source

CE-MR scans were performed using 3 types of machines: 1.5-T MRI scanners (Aurora Imaging Technology, Aurora Systems, Inc., Canada and GE, Signa HDx) and a 3.0-T MRI scanner (Siemens Healthineers, Erlangen, Germany) with a 16-channel body coil. All patients were scanned in the supine position. The sequences and MR scanning parameters are listed in the Additional file 1.

Regions of interest (ROIs) were placed semiautomatically on the peak enhanced phase of CE-MRI by 3D Slicer software (https://www.slicer.org/) by one radiologist with 15 years of experience in breast imaging. ROIs were placed on all slices that contained the whole tumor and the largest lesion (in the case of multicentric or multifocal tumors). The radiologist was blinded to the patients’ clinical and pathological information.

### Radiomic feature extraction

This study extracted the radiomic features of the contrast-enhanced phase using the PyRadiomics version 2.1.2 (https://pyradiomics.readthedocs.io/en/latest/features.html). Feature extraction was performed in the tumoral and peritumoral regions. The peritumoral region was delineated by expanding the tumor outward with a 2-pixel width and subtracting the tumor area. Three groups of radiomics features were extracted in this study: first order features, wavelet features and texture features including gray-level dependence matrix (GLDM) features, gray-level cooccurrence matrix (GLCM) features, gray-level size-zone matrix (GLSZM) features, gray-level run-length matrix (GLRLM) features, and neighborhood gray tone difference matrix (NGTDM) features.

Radiomic features were extracted from baseline and follow-up images after 2 NAC cycles and were used to build the baseline radiomic (radiomics-baseline) model and radiomic model after 2 cycles (radiomics after 2 cycles). The features were used as attributes for the LightGBM classifier, and the extracted features remained the same across the three types of MRI machines used.

### Radiomic data analyses

After Z-score standardization, we concatenated the features of the three types of MRI machines. Principal component analysis was first performed to ensure that all the radiomic features were distributed evenly among the 3 machine types (Additional file 1: Figure S1) and could be used in further analyses. Then, we randomly divided the dataset into a training set and a validation set at a ratio of 2:1. Finally, we recorded the user_id of the patients to split the genomic data into the same datasets.

**Fig. 2:**
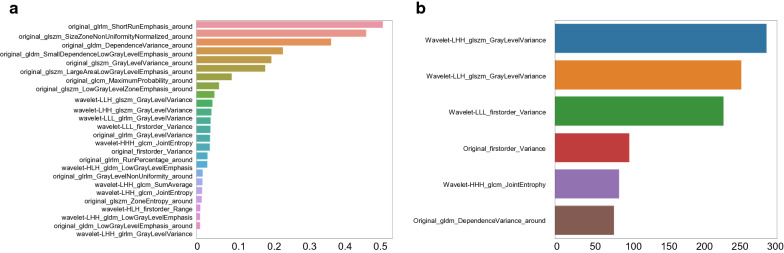
Details of radiomic feature extraction using LASSO (**a**) and XGBoost (**b**) at baseline. Two feature selection steps were applied to the extracted radiomic features with the least absolute shrinkage and selection operator (LASSO) and XGBoost. **a** The LASSO model is a linear combination of the selected features weighted by their respective coefficients. The x-axis denotes LASSO coefficients. Features with nonzero coefficients denote greater contributions to the model and were selected. **b** Feature importance evaluates how valuable each feature is in the construction of the gradient boosted decision trees within the XGBoost model and is calculated by information gain. The x-axis measures the information gain

After all the preprocessing steps of pretreatment mentioned above, least absolute shrinkage and selection operator (LASSO) regression and XGBoost were used in turn to screen the radiomics features both at baseline and after 2 cycles. Variables with a high coefficient were associated with the response to NAC by using LASSO (Fig. 2a and Additional file 1: Figure S2a). Next, XGBoost was applied to further select features for model establishment. Finally, we selected 6 radiomic features from the data obtained at baseline and after 2 cycles (Fig. 2b and Additional file 1: Figure S2b).

LightGBM models were built to predict pCR in the training set, and hyperparameters were selected, including the number of leaves, minimum data in leaves, maximum depth and learning rate via fivefold cross-validation. We identified the optimal parameter combination according to the performance during cross-validation and applied this to the final model using the best parameter combination in the validation set. Receiver operating characteristic (ROC) curves were constructed.

### Genomic feature extraction

Both fresh tumor tissues (obtained using baseline core-needle biopsy) and peripheral blood samples were collected. Total DNA was isolated, quantified and processed as previously reported [17]. Qualified genomic DNA from both tissues and the matched white blood cell samples was sequenced using the FUSCC-BC panel,.

Data were collected using Illumina Real Time Analysis (RTA) and assembled into FASTQ files using Illumina Bcl2Fastq2. The high-quality reads were mapped to the hg19 version of the human reference genome (GRCh37) using the BWA aligner with the BWA-MEM algorithm and default parameters. The Genome Analysis Toolkit (GATK) was used to locally realign the BAM files at intervals with mismatched indels and recalibrate the base quality scores of the reads in the BAM files. Somatic mutations were called from the tissue and blood BAM files using GATK4 Mutect2 with the default parameters. The VCF files were annotated using ANNOVAR. To improve specificity, a panel of normal sample filters was used to filter the expected germline variations and artifacts [15]. Each alteration identified by the pipeline was manually reviewed to ensure that no false positives were reported. The sequencing quality statistics were obtained using SAMtools and GATK. The FACETS algorithm [19] was utilized to detect gene-level amplification and deletion. Characterization of Germline Variants (CharGer) [20] was used to further classify germline variants. Further details on sample preparation and sequencing data generation can be found in our previous work [17].

### Radiogenomic data analyses

The somatic mutation data included in this study were annotated and visualized using Maftools in R version 3.6.2 Pearson’s chi-square test was employed for comparison of unordered categorical variables.

For model prediction, we first summed the variant allele frequency (VAF) values of the nonsynonymous mutation sites in each gene. The Z-score was used to standardize these features and the features were selected by XGBoost. All features with an information gain over 75 were selected. The selected 5 genomic features and the 6 abovementioned radiomic features were used for the radiogenomic model. The training and validation datasets were the same as those in the radiomic analyses. Fivefold cross-validation was utilized to obtain the best hyperparameters with the highest AUC as the final model, and we tested this model on the validation set.

### Framework of drug sensitivity validation for the two recurrent mutations

The mutation profiles of pCR and non-pCR populations were compared. Among them, the *REL* and *MED23* mutations were significantly different between the groups, and all the mutations were present in the non-pCR population. Each of these two genes had one high-frequency mutation, MED23 p.P394H and REL p.D268E. Thus, we further explored the two recurrent mutations with in vitro drug assays. Epirubicin and paclitaxel, two well-accepted and widely-used drugs for TNBC were applied to assess drug resistance after mutation.

The detailed methods for the in vitro experiments, including the cell lines used and culture conditions, plasmid construction, western blot analysis, IC50 assays, colony formation survival, apoptosis analysis, immunofluorescence and homologous recombination (HR) DNA repair assay are included in the Additional file 1. All experiments were repeated three times under the same conditions. Detailed information on the expression constructs and primers is provided in Additional file 1: Table S1. Detailed information on the antibodies used in this study is summarized in Additional file 1: Table S2.

### Statistical analysis

Continuous variables are reported as the median and interquartile range (IQR) and were compared using the Wilcoxon signed-rank test. Statistical significance was defined as *p* < 0.05. Genomics and radiomics analyses were performed using the LightGBM 2.2 package, Python version 3.7.0 (https://www.python.org/downloads/), whereas comparisons of basic benchwork data were performed using SPSS 22.0 software. The DeLong test was applied to compare the area under the curve (AUC) values between different predictive models.

For experimental results, all data are presented as the means ± SDs and represent at least three independent experiments. The unpaired two-tailed Student’s t test was used to compare data between two groups. One-way analysis of variance was used to compare the means between treatment groups. The Mann–Whitney Wilcoxon test and the Kruskal–Wallis test were utilized to compare ordered categorical variables. A *p* value < 0.05 was considered statistically significant.

## Results

### Patient characteristics

From April 2018 to September 2019, 127 consecutive women with TNBC who underwent NAC and participated in the Precision Oncology Program for Exome Sequencing were included. Among them, 112 met the eligibility criteria and had complete endpoint data (Fig. 1). The baseline patient characteristics according to pCR status are listed in Table 1.

**Table 1:** Characteristics of the study population

	pCR (%)	Non-pCR (%)	Whole population (%)
N	28 (25.0)	84 (75.0)	112
Median age	47.0	51.5	50.5
IQR	39.0–55.0	39.0–59.0	39.0–58.0
Menopausal status [n (%)]			
Premenopausal	17 (60.7)	41 (48.8)	58 (51.8)
Postmenopausal	11 (39.3)	43 (51.2)	54 (48.2)
Baseline clinical staging [n (%)]			
Stage I	1 (3.6)	1 (1.2)	2 (1.8)
Stage II	13 (46.4)	34 (40.5)	47 (42.0)
Stage III	14 (50.0)	49 (58.3)	63 (56.3)
Median overall NAC cycles (IQR)	8.0–8.0	5.0–8.0	6.0–8.0
NAC regimens [n (%)]			
Anthracycline-and-Taxane-based	25 (89.3)	65 (77.4)	90 (80.4)
Anthracycline-based only	0 (0)	8 (9.5)	8 (7.1)
Taxane-based only	3 (10.7)	11 (13.1)	14 (12.5)
Breast surgery			
Breast conserving surgery	5 (17.9)	12 (14.3)	17 (15.2)
Mastectomy	23 (82.1)	72 (85.7)	95 (84.8)
Axillary surgery			
SLNB	5 (17.9)	10 (11.9)	15 (13.4)
ALND	23 (82.1)	74 (88.1)	97 (86.6)

In total, 25.0% of the patients achieved pCR after completing all NAC cycles. The proportions of patients with pCR and non-pCR were balanced between the training and validation sets. Approximately 52% of the population was premenopausal, and the median age was 50.5 years. Most patients (98.2%) presented with stage II-III disease. A total of 87.5% of the patients received NAC regimens containing anthracyclines, whereas the remaining 12.5% were administered taxane-based regimens only.

### Baseline radiomic features predict pCR

The top ranked radiomic features in the training set with reliability based on multiple-sequences are shown in Fig. 2 (baseline) and Additional file 1: Figure S2 (after 2 cycles). Furthermore, 6 baseline radiomic features and 6 features after 2 cycles were selected for the classifier.

The baseline radiomic model predicted pCR well, with an AUC of 0.71 (95% CI 0.61–0.81). In the validation set, the performance was still stable, with an AUC of 0.73 (95% CI 0.66–0.82).

Compared with that of the baseline model, the AUC value of the radiomic model after 2 cycles was not significantly different (training set: AUC 0.71 vs. 0.73, *p* = 0.89; validation set: AUC 0.73 vs. 0.69, *p* = 0.55. See Additional file 1: Figure S3). The predictive value of the baseline MR radiomic model was not significantly worse than that of the model after 2 cycles, which illustrates the early and accurate predictive ability of baseline MR radiomic features.

### Somatic genomic alterations in the study population

In the study population, 5880 somatic mutations were identified, comprising 5442 single-nucleotide variants (SNVs) and 438 insertions or deletions (INDELs). These tumors harbored a median of 45 nonsynonymous SNVs and 4 INDELs. The mutation profile is shown in Additional file 1: Figure S4. The most prominent cancer-related variations observed in this cohort were *TP53* mutations (66%), followed by *PIK3CA* mutations (21%), whereas other mutations occurred in less than 10% of the cohort. Comparing the mutation profile of pCR and non-pCR populations, the top 8 mutations are listed in Table 2. Among them, the *REL* (*p* = 0.035) and *MED23* (*p* = 0.036) mutations were significantly different between the two groups, and all mutations were present in the non-pCR population.

**Table 2:** Different mutations between the pCR and non-pCR populations (top 8 ranking genes)

Gene	pCR (n = 28)	Non-pCR (n = 84)	*p*
*REL*	0	9	**0.035**
*MED23*	0	8	**0.036**
*PIK3CA*	3	20	0.182
*PTEN*	4	5	0.219
*AKT1*	1	7	0.677
*TP53*	19	55	0.815
*ATR*	1	5	1.000
*KMT2C*	2	9	1.000

*p* values < 0.05 are shown in bold

### Radiogenomic model predicts pcr better than the radiomic model

**Fig. 3:**
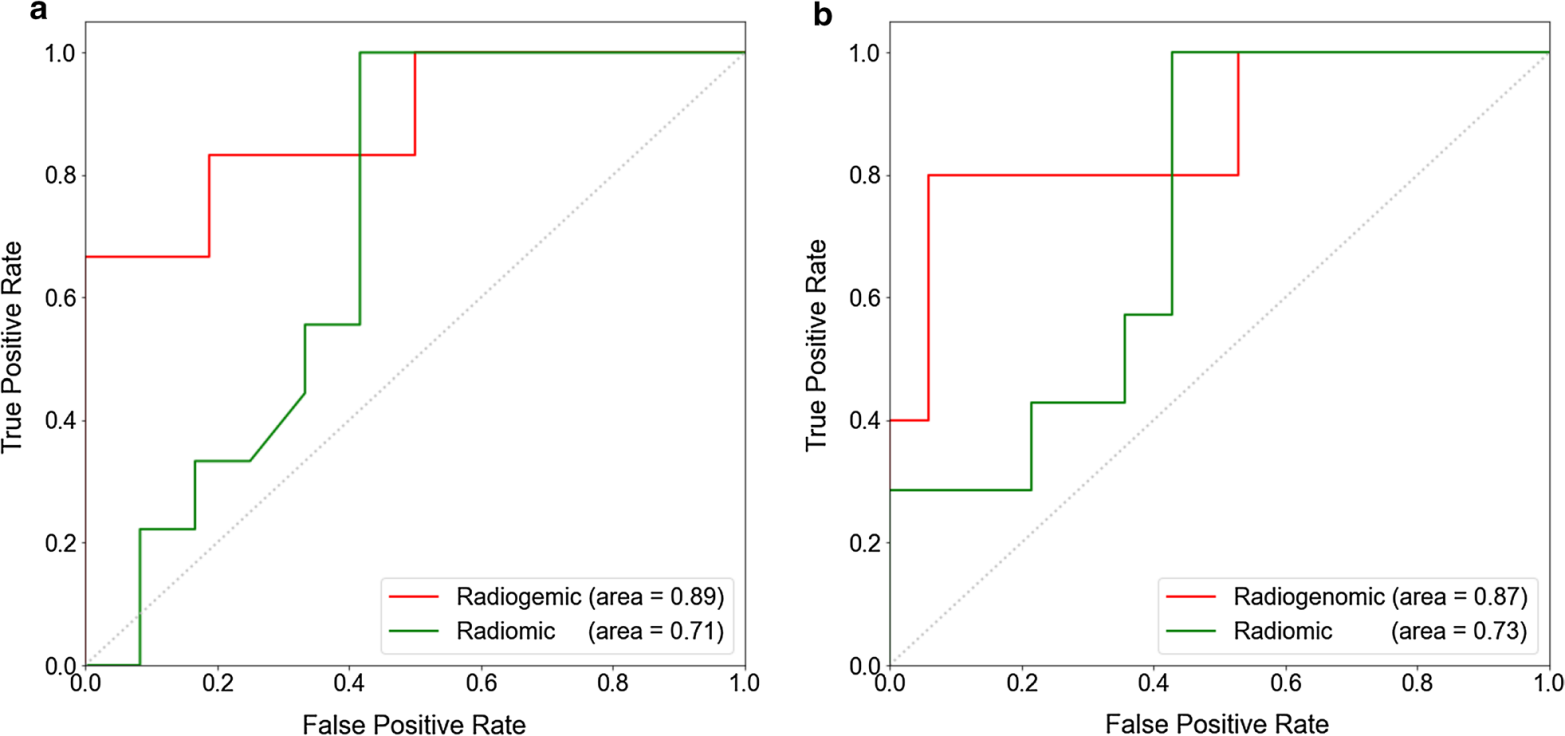
ROC curves of the radiogenomic (red) and radiomic models (green). **a** Training set. **b** Validation set

XGBoost was used to select 5 VAF features that might help to determine pCR status, namely, *TEC*, *PIK3CA*, *REL*, *MAPK10* and *MED23* (Additional file 1: Figure S5). The radiogenomic model was overwhelmingly superior to the baseline radiomic model in differentiating pCR from non-pCR, with an AUC of 0.89 (95% CI 0.74–0.95) vs. 0.71 (95% CI 0.61–0.81, *p* = 0.024). This finding was further supported by the results from the validation set [radiogenomic versus radiomic model: 0.87 (95% CI 0.73–0.91) vs. 0.73 (95% CI 0.66–0.82), respectively; p = 0.039] (Fig. 3).

### MED23 p.P394H weakened epirubicin sensitivity through HR repair

**Fig. 4:**
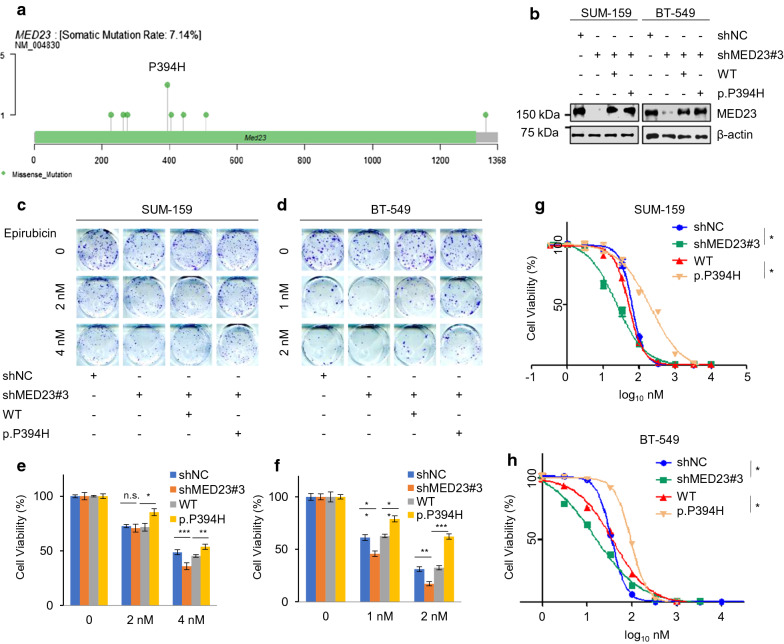
IC_50_ and colony formation assay with epirubicin treatment in stable cells expressing wild-type MED23 and P394H mutation. **a** MED23 missense mutations discovered in this cohort. MED23 p.P394H was identified as a recurrent mutation. **b** MED23 was knocked down via shRNA. SUM-159 and BT-549 cells stably expressing wild-type MED23 or p.P394H mutation were subjected to immunoblotting. **c–f** SUM-159 and BT-549 cells stably expressing wild-type MED23 and P394H mutation were treated with increasing doses of epirubicin and subjected to colony formation survival assays. Representative images of the surviving colonies are shown in **C** and **D**, and the corresponding quantitative results are shown in **E** and **F**. **g**, **h** SUM-159 and BT-549 cells stably expressing wild-type MED23 and P394H mutation were treated with increasing doses of epirubicin and subjected to IC_50_ assays. *p < 0.05, **p < 0.01, ***p < 0.001

Mutations in MED23 and REL were significantly more common in the non-pCR population. Each of these two genes had one high-frequency mutation (recurrent spot) in our cohort, namely, MED23 p.P394H and REL p.D268E, which occurred in 3 and 2 patients, respectively (Fig. 4a and Additional file 1: Figure S6a). Thus, we further explored whether the two high-frequency mutations play a role in drug resistance.

We first generated stable cell lines carrying wild-type (WT) MED23 and MED23 p.P394H in MED23 knockdown SUM-159 and BT-549 cells (Fig. 4b and Additional file 1: Figure S7). The cell lines were treated with increasing doses of epirubicin and paclitaxel and subjected to functional assays. Compared with MED23-WT cells, cells expressing the P394H mutant protein showed stronger viability in the colony formation assay after treatment with epirubicin, indicating that P394H-mutant cells may be resistant to epirubicin (Fig. 4c–f). As expected, the IC50 of the mutant cells was also higher than that of the wild-type cells (WT vs. p.P394H: SUM-159 208.7 vs. 51.52 nmol, *p* < 0.05; BT-549 94.56 vs. 37.64 nmol, *p* < 0.05, Fig. 4g and h). In contrast, neither the cell viability nor IC50 of these two cell lines were significantly different when the cells were treated with paclitaxel (Additional file 1: Figure S8). Thus, we infer that the drug resistance related to MED23 p.P394H might be anthracycline-specific. The results of further apoptosis analysis showed a similar trend (Additional file 1: Figure S9).

**Fig. 5:**
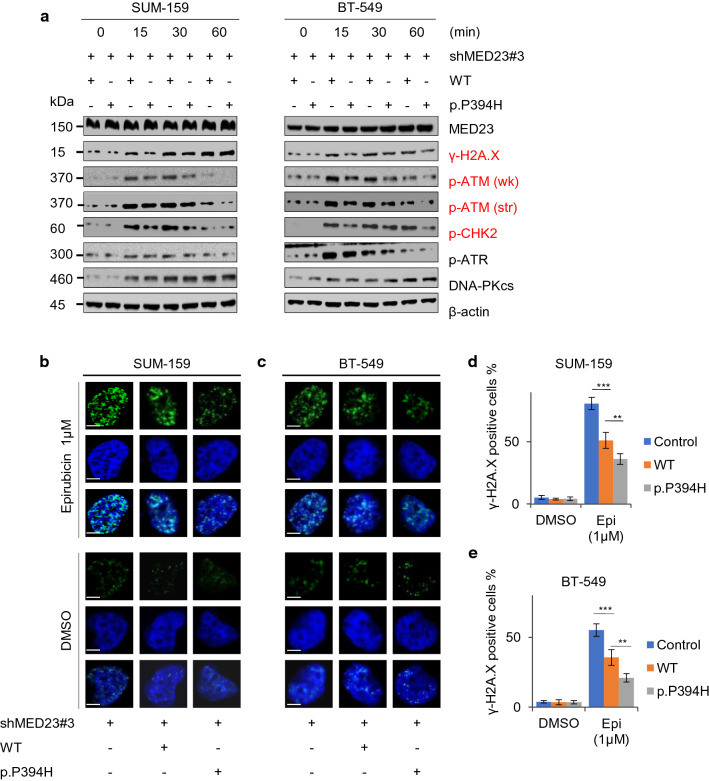
MED23 p.P394H mutation might cause epirubicin resistance by promoting DNA damage repair by affecting the p-ATM- γ-H2A.X- p-CHK2 pathway. **a** Stable SUM-159 and BT-549 cells expressing wild-type MED23 and the P394H mutation were treated with epirubicin (100 nM for 0,15,30 and 60 min) and then subjected to immunoblotting for various DNA damage markers. **b**–**e** Stable SUM-159 and BT-549 cells expressing wild-type MED23 or P394H were treated with or without 1 μM epirubicin for 4 h and then stained with anti-γ-H2A.X antibody (green). Cell nuclei were counterstained with DAPI (blue). Quantitative results of γ-H2A.X-positive cells (the number of foci > 15 per cell) are shown in **C** and **D**. ***p < 0.001, **p < 0.01. Scale bar, 5 μm

Next, we treated the cells with 100 nM epirubicin for 0, 15, 30 min and 1 h and found that the expression of the DNA damage marker γ-H2A.X was significantly downregulated in P394H mutant cells (Fig. 5a). Meanwhile, its upstream protein p-ATM and downstream protein p-CHK2 presented the same trend. Moreover, the results of the immunofluorescence assay were consistent with those of western blot analysis (Fig. 5b-e).To further explain the epirubicin resistance induced by P394H mutation, we investigated whether HR repair was involved. In the DR-GFP reporter assay [21], P394H-mutant U2OS cells exhibited a significant increase (3.0% vs. 2.4%, *p* < 0.01) in the percentage of GFP-positive cells compared with WT cells, demonstrating that MED23 p.P394H mutation enhanced HR (Additional file 1: Figure S10a and c). These results collectively indicated that P394H may lead to epirubicin resistance by enhancing HR repair via the p-ATM- γ-H2A.X- p-CHK2 pathway.

The other high-frequency mutation, REL p.D268E showed no significant effects on cell viability or IC50 values, regardless of whether the cells were treated with epirubicin or paclitaxel (Additional file 1: Figure S6).

## Discussion

TNBC is characterized by its high rate of early recurrence and poor survival, with limited treatment options available [22]. In the neoadjuvant setting, pCR remains the most convincing substitute indicator of survival [2]. For potential non-pCR patients with an increased risk of chemoresistance, treatment modification (e.g., the addition of immunotherapy [23], nab-paclitaxel [24, 25] or nanoparticles for codelivery of antitumoral agents [26]) might be an option to increase the possibility of pCR. In this study, we aimed to establish a radiogenomic model using LightGBM to predict the treatment response as early as possible.

Quantitative imaging features that can be used to predict the response to treatment can expand the application of radiomics in routine clinical settings [11]. Braman et al. [27] proposed that both intratumoral and peritumoral features can contribute to response predictions, and that peritumoral features cannot be replaced by tumoral features. Therefore, we also included peritumoral radiomic features to avoid missing information from the surrounding microenvironment. The selected radiomic features, including first-order_variance, GLDM and GLCM parameters were consistent with those in other studies [10, 28–30]. Here we newly identified a series of wavelet-related features that also contributed to pCR prediction. Wavelet features have been previously mentioned in the response prediction of colorectal cancer [31], but their application in breast cancer treatment response remain unexplored., which is worthy of further investigation. Moreover, the addition of clinicopathological data has been reported to increase the predictive ability compared to that of the model alone [32], but external validation has failed to confirm the superiority of the radiomic model over the clinical model [29]. In our study, we also failed to show a significant improvement in pCR prediction with the addition of clinical factors. Studies with larger sample sizes are needed to justify the addition of clinicopathological features into a radiomic/radiogenomic model. In the ACRIN 6657/I-SPY study [33], prediction based on changes in tumor volume showed the greatest relative benefit after 1 cycle of anthracycline-based NAC, with an AUC of 0.70. Another recent study [30] revealed excellent predictive power for 3-year recurrence (AUC = 0.93) in TNBC, but both pre- and post-NAC features were used. Our radiogenomic model enabled the prediction of pCR even before the start of NAC (at baseline), with a high AUC value of 0.87 in the validation set, thus potentially supporting earlier treatment modification. Patients with a poor response might not need to complete additional cycles prior to treatment alteration.

In addition to the macroscopic and high-throughput characteristics from radiology, we tried to add genomic data to enhance the predictive value, discover novel biomarkers and identify potential genomic mechanisms associated with the drug-resistant phenotype. For response predictions, our results revealed the excellent performance of the radiogenomic model after the addition of 5 VAF features, with an AUC of To identify potentially actionable targets, we tested whether the two high-frequency mutation spots (MED23 p.P394H and REL p.D268E) were associated with chemotherapy resistance. Mediator (MED) is a multisubunit complex that conveys signals to the basal transcription machinery [34]. Mediator complex subunit 23 (MED23) is a subunit that plays a crucial role in alternative mRNA processing [35], cell differentiation [36] and tumorigenesis [37]. Overexpression of MED23 was confirmed to promote tumorigenesis in NSCLC [38] and hepatocellular carcinoma [39]. Conversely, overexpression of MED23 in esophageal squamous cell carcinoma dramatically inhibited cell growth [40]. However, few studies have investigated the role of MED23 in breast cancer. We found that the cell viability and IC50 values of MED23 p.P394H-mutant cells increased after epirubicin treatment. This trend was opposite to that observed after MED23 was knocked down, indicating that P394H might be a pathogenic mutation.

Epirubicin forms complexes with DNA by intercalation between base pairs, which leads to the formation of free radicals and inhibits the catalytic activity of DNA topoisomerase II [41]. Conversely, DNA damage repair is an important contributor to epirubicin resistance [42]. Significantly reduced levels of p-ATM and γ-H2A.X and p-CHK2 were observed at multiple time points post-mutation, indicating that epirubicin resistance after P394H mutation might manifest through regulation of the p-ATM- γ-H2A.X- p-CHK2 pathway. HR is one of the most important mechanisms for the repair of anthracycline-DNA adducts [43]. Our results showed that the P394H mutation might affect HR repair, thus further inducing epirubicin resistance. Cancer cells with efficient DNA damage repair machinery might be able to overcome the cytotoxicity of anthracyclines. Further experiments are needed to explain the underlying mechanisms and verify whether MED23 p.P394H or HR inhibitors could help to reverse epirubicin resistance. Another high-frequency mutation, REL p.D268E, failed to elicit any significant effect on drug resistance, possibly because of the limited sample size. The relatively high-frequency (2 patients) of this mutation in this cohort might not reflect its true frequency in the general TNBC population.

Despite the excellent predictive value of the radiogenomic model, several limitations still need to be considered. First, actionable somatic mutations are not frequent events in TNBC patients, so bias existed in this relatively small cohort. Further validation in external populations is needed. Additionally, the epirubicin-resistant phenotype caused by MED23 P394H and its underlying mechanisms need to be fully explored in clinical samples in the future. Second, this was a single-institution study, the chemotherapy regimens were not unified, and heterogeneity existed due to the use of different MRI machines. Third, the genomic data were sequenced using core-needle biopsy tissue, which might be affected by paracancerous tissue. Finally, the genomic analyses only covered mutation data; other aspects, including copy number variation and quantitative expression, were not included.

Despite these limitations, in this work, an excellent radiogenomic model was constructed, with an AUC as high as 0.87, that could predict pCR in the TNBC population prior to NAC administration. Furthermore, we are the first to propose that the MED23 p.P394H mutation might cause epirubicin resistance and to explore this phenotype. The underlying mechanisms will be investigated and validated in future experiments.

## Conclusions

The proposed radiogenomic model has the potential to accurately predict pCR before NAC in TNBC patients. The resistance to epirubicin observed after MED23 p.P394H mutation occurs might be associated with HR repair through regulation of the p-ATM-γ-H2A.X-p-CHK2 pathway.

## Supplementary Information

**Additional file 1: Figure S1.** Principal component analyses of radiomic features from 3 types of MRI machines. **Figure S2.** Details of radiomic feature extraction using LASSO (a) and XGBoost (b) after 2 cycles. Two feature selection steps were applied to the extracted radiomic features with the least absolute shrinkage and selection operator (LASSO) and XGBoost. (a) The LASSO model is a linear combination of the selected features weighted by their respective coefficients. The x-axis denotes LASSO coefficients. Features with nonzero coefficients denote greater contributions to the model and are selected. (b) Feature importance evaluates how valuable each feature was in the construction of the gradient boosted decision trees within the XGBoost model and is calculated by information gain. The x-axis measures the information gain. **Figure S3.** ROC curves of the radiomic model at baseline (green) and after 2 cycles (yellow). **Figure S4.** Mutation profile oncoplot of the study population. **Figure S5.** Selection of genomic features using XGBoost. For genomic feature selection, variables including mutation status (positive or negative), detected mutation counts and VAF of each gene were input. After standardization, feature importance evaluates how valuable each feature was in the construction of the gradient boosted decision trees within the XGBoost model and is calculated by information gain. The x-axis measures the information gain. Features with the top 10 information gain rankings are presented. The selection criterion was defined as an information gain over 75. Therefore, 5 VAF features were selected. **Figure S6.** IC50 and colony formation assay with epirubicin and paclitaxel treatment in stable cells expressing wild-type REL and D268E mutation. (a) REL mutations discovered in this cohort. REL p.D268E was identified as a recurrent mutation. (b) Stable shREL SUM-159 and MDA-MB-231 cells were further r transfected with wild-type REL or p.D268E mutation, respectively, and subjected to immunoblotting. (c-f) SUM-159 and MDA-MB-231 cells stably expressing wild-type REL and the D268E mutation were treated with increasing doses of epirubicin and subjected to colony formation survival assays. Representative images of the surviving colonies are shown in C and D, and the corresponding quantitative results are shown in E and F. (g-h) SUM-159 and MDA-MB-231 cells stably expressing wild-type REL and the D268E mutation were treated with increasing doses of epirubicin and subjected to IC50 assays. *p< 0.05, **p< 0.01, ***p< 0.001 (i-l) SUM-159 and MDA-MB-231 cells stably expressing wild-type REL and D268E mutation were treated with increasing doses of paclitaxel and subjected to colony formation survival assays. Representative images of surviving colonies are shown in I and J, and the corresponding quantitative results are shown in K and L. (m-n) SUM-159 and MDA-MB-231 cells stably expressing wild-type REL and the D268E mutation were treated with increasing doses of paclitaxel and subjected to IC50 assays. *p< 0.05, **p< 0.01, ***p< 0.001 **Figure S7.** Construction of MED23 (a) and REL (b) knockdown cell lines. (a) SUM-159 and BT-549 cells were transfected with shMED23 and shNC. After selection by puromycin, the cells were collected and subjected to immunoblotting. (b) SUM-159 and MDA-MB-231 cells were transfected with shREL and shNC. After 48 h of transfection, the cells were treated with puromycin for 7-10 days at a concentration of 5 μg/ml and then subjected to immunoblotting. **Figure S8.** IC50 and colony formation assay with paclitaxel treatment in stable cells expressing wild-type MED23 and P394H mutation. (a-d) MED23 was knocked down via shRNA. SUM-159 and BT-549 cells stably expressing wildtype MED23 and P394H mutation were treated with increasing doses of paclitaxel and subjected to colony formation survival assays. Representative images of surviving colonies are shown in A and C, and the corresponding quantitative results are shown in B and D. (e-f) SUM-159 and BT-549 cells stably expressing shNC, shMED23, wild-type MED23 and P394H mutation were treated with increasing doses of paclitaxel and subjected to IC50 assays. *p< 0.05, **p< 0.01, ***p< 0.001. **Figure S9.** Apoptosis of SUM-159 and BT-549 cells stably expressing wild-type MED23 and p.P394H mutation after epirubicin treatment. The upper right quadrant represents dead/late apoptotic cells, whereas the lower right quadrant represents early apoptotic cells. After treatment with epirubicin (10 nM, 24 h), cells were stained with Annexin V/PE and 7AAD, and observed by flow cytometry. *p< 0.05, **p< 0.01, ***p< 0.001. **Figure S10.** MED23 p.P394H promoted homologous recombination repair. (a) U2OS cells expressing wild-type MED23 and the P394H mutation were transfected with I-SceI. After 24 h, the cells were treated with 10 μM triamcinolone acetonide for another 48 h. The HR reporter assay was determined by flow cytometry. (b) The working model of the HR reporter system is presented. I-SceI endonuclease was introduced into GFP/U2OS cells. (c) Quantification of GFP-positive cells. **p< 0.01 **Table S1.** Detailed information on the expression constructs (a), primers (b) and shRNA sequences (c). **Table S2.** The vendors and working concentrations of antibodies.

## Data Availability

The datasets used and/or analyzed during the current study are available from the corresponding author upon reasonable request.

## Abbreviations

pCR: Pathological complete response
CE-MRI: Contrast-enhanced magnetic resonance imaging
LightGBM: Light gradient boosting machine
VAF: Variant allele frequency
TNBC: Triple-negative breast cancer
NAC: Neoadjuvant chemotherapy
AUC: Area under the curve
AI: Artificial intelligence
ROI: Regions of interest
GLDM: Gray-level dependence matrix
GLCM: Gray-level cooccurrence matrix
GLSZM: Gray-level size-zone matrix
GLRLM: Gray-level run-length matrix
NGTDM: Neighborhood gray tone difference matrix
LASSO: Least absolute shrinkage and selection operator
MAF: Mutation annotation format
IQR: Interquartile range
SNV: Single-nucleotide variant
DSB: Double-strand break
HR: Homologous recombination
MED23: Mediator complex subunit 23
