# Supplementary material for: Integration of radiogenomic features for early prediction of pathological complete response in patients with triple-negative breast cancer and identification of potential therapeutic targets
Source: J Transl Med. 2022 Jun 7;20:256. doi: 10.1186/s12967-022-03452-1 (PMC9171937; doi:10.1186/s12967-022-03452-1)
Supplement: Supplementary file 1 — Additional file 1: Figure S1. Principal component analyses of radiomic features from 3 types of MRI machines. Figure S2. Details of radiomic feature extraction using LASSO (a) and XGBoost (b) after 2 cycles. Two feature selection steps were applied to the extracted radiomic features with the least absolute shrinkage and selection operator (LASSO) and XGBoost. (a) The LASSO model is a linear combination of the selected features weighted by their respective coefficients. The x-axis denotes LASSO coefficients. Features with nonzero coefficients denote greater contributions to the model and are selected. (b) Feature importance evaluates how valuable each feature was in the construction of the gradient boosted decision trees within the XGBoost model and is calculated by information gain. The x-axis measures the information gain. Figure S3. ROC curves of the radiomic model at baseline (green) and after 2 cycles (yellow). Figure S4. Mutation profile oncoplot of the study population. Figure S5. Selection of genomic features using XGBoost. For genomic feature selection, variables including mutation status (positive or negative), detected mutation counts and VAF of each gene were input. After standardization, feature importance evaluates how valuable each feature was in the construction of the gradient boosted decision trees within the XGBoost model and is calculated by information gain. The x-axis measures the information gain. Features with the top 10 information gain rankings are presented. The selection criterion was defined as an information gain over 75. Therefore, 5 VAF features were selected. Figure S6. IC50 and colony formation assay with epirubicin and paclitaxel treatment in stable cells expressing wild-type REL and D268E mutation. (a) REL mutations discovered in this cohort. REL p.D268E was identified as a recurrent mutation. (b) Stable shREL SUM-159 and MDA-MB-231 cells were further r transfected with wild-type REL or p.D268E mutation, respectively, and subjected [file 12967_2022_3452_MOESM1_ESM.docx]

Additional file 1

**Sequences and MR Scanning Parameters:**

(a) For the Siemens system, the sequences included diffuse-weighted imaging (b= 1000 s/mm^2^, time of repetition (TR)/time of echo (TE) = 5600/75 ms, flip angle= 90°, field of view (FOV) = 180 × 300 mm^2^, slice thickness= 5 mm, slice gap= 0, band width=1666 Hz, EPI= 96) and dynamic contrast-enhanced sequences (TR/TE= 5.1/1.7 ms, flip angle= 15°, FOV = 260 × 260 mm^2^, slice thickness= 3 mm, slice gap= 0). The contrast agent Gd-DTPA (0.1 mmol/kg) was infused at a flow rate of 3.0 mL/s, followed by flushing with 15 mL of saline. Images from the precontrast phase and six contrast phases were collected.

(b) For the Aurora system, the sequences included axial T2-weighted turbo spin-echo sequences with fat suppression (T2-weighted imaging, T2WI; TR= 6680 ms, TE= 68 ms, slice thickness = 3 mm, slice gap = 1 mm), T1-weighted gradient echo (T1-weighted imaging, T1WI; TR= 5 ms, TE= 13 ms, slice thickness = 3 mm, slice gap = 1 mm), and dynamic contrast–enhanced T1-weighted sequences with fat plus water suppression sequences (TR= 5 ms, TE= 29 ms, slice thickness= 1.1 mm, slice gap= 0, FOV= 360×360 mm^2^). The contrast agent Gd-DTPA (0.2 mmol/kg) was infused at 90 s of the precontrast phase at a flow rate of 2.0 mL/s, followed by flushing with 20 mL of saline. Images from the precontrast phase and four contrast phases were collected. The acquisition time was 180 s.

(c) For the GE system, the sequences included axial T2-weighted turbo spin-echo sequences with fat suppression (T2-weighted imaging, T2WI, TR= 6680 ms, TE= 68 ms, slice thickness = 3 mm, slice gap = 1 mm), T1-weighted gradient echo (T1-weighted imaging, T1WI; TR= 5 ms, TE= 13 ms, slice thickness = 3 mm, slice gap = 1 mm), and dynamic contrast–enhanced T1-weighted sequences with fat plus water suppression sequences (TR= 6.5 ms, TE= 3.5 ms, slice thickness= 3 mm, slice gap= 0, FOV 300×300 mm^2^). The contrast agent Gd-DTPA (0.2 mmol/kg) was infused at 90 s of the precontrast phase at a flow rate of 2.0 mL/s, followed by flushing with 20 mL of saline. Images from the precontrast phase and four contrast phases were collected. The acquisition time was 161 s.

**Drug Sensitivity Validation and the Potential Phenotypes of the two Selected Recurrent Mutation Sites.**

***Cell Lines and Culture***

The human embryonic kidney cell line (HEK293T), human breast cancer cell lines (MDA-MB-231 and BT-549) and osteosarcoma cell line (U2OS) were obtained from the Shanghai Cell Bank Type Culture Collection Committee (CBTCCC; Shanghai, China) in 2014, whereas the human breast cancer cell line (SUM-159) was obtained from Prof. Suling Liu (Fudan Institutes of Biomedical Sciences Laboratory, Shanghai). All the cell lines were authenticated by mycoplasma detection, DNA-fingerprinting, isozyme, and cell vitality. The cell lines were expanded and frozen immediately into numerous aliquots after arrival. The cells revived from the frozen stock were used within 10-15 passages and 6 months.

The cells were cultured in DMEM (Basal Media, #L110) supplemented with 10% fetal bovine serum (FBS) (Gibco, #10270-106) and 1% penicillin/streptomycin (Basal Media, #S110B).

***Plasmid Construction****.*

The Flag-MED23 (#22486GR-P) and Flag-MED23 p.P394H (#22486GS-P) expression constructs were purchased from Genomeditech, Shanghai. The shMED23 plasmid (#89454-89456) and REL-blast (#GOSE0240802) expression constructs were purchased from GeneChem, Shanghai. Short hairpin RNA (shRNA) sequences targeting human REL were obtained from BLOCK-iT RNAi Designer (http://rnaidesigner.thermofisher.com/rnaiexpress/) and then cloned into the pLKO.1-TRC vector (#10878, Addgene). The REL p.D268E mutation was generated using the ClonExpress Ultra One Step Cloning Kit (Vazyme, #C115-02) and CloneEZ PCR Cloning Kit (Genscript, #L00339). All the constructs were veriﬁed by sequence analysis (Sangon Biotech).

Detailed information on the expression constructs and primers is provided in **Table S1**. Stable plasmid transfection was performed using DNA transfection reagent (Teye Biotechnology, #FT19301) according to the manufacturer’s recommended protocol. To generate stable cell lines, cells were transfected with each lentivirus expression vector and packaging plasmid mix using DNA transfection reagents. The supernatant containing the virus was collected 48 h after transfection, ﬁltered, and used to infect the target cells in the presence of 8 µg/ml of polybrene (Sigma, #H9268) before drug selection with 5 µg/ml of puromycin ± 5 µg/ml of blasticidin for 1 week.

***Western Blot Analysis.***

Whole-cell lysates were resolved using T-PER Tissue Extraction Reagent (Thermo Fisher Scientiﬁc Inc., #78510) with a complete ethylenediaminetetraacetic acid-free protease inhibitor and phosphatase inhibitor cocktail (Bimake Chemicals, #B15003). Immunoblotting was performed using a standard method. Detailed information on the antibodies used in this study is provided in **Table S2**. The quality of the gel loading and transfer processes was assessed by immunostaining the blots using the internal controls vinculin and β-actin.

***IC50 Assays.***

For the IC50 assays, 2 × 103 cells in the logarithmic growth stage were plated in 96-well plates. Epirubicin (#S1223) and paclitaxel (#1150) were purchased from Selleck Chemicals. The cells were allowed to adhere overnight, and then the medium was replaced with medium containing serially diluted concentrations of drugs for 72 h. Ten microliters of CCK-8 solution (Yeason, #40203ES60) was added to each well, the plates were incubated for 4 h, and absorbance at 450 nm was determined. The IC50 was calculated using GraphPad Prism.

***Colony Formation Survival***

A total of 1× 103 cells were seeded into 6-well plates in triplicate overnight, and treated with the indicated drugs. The cells were fixed with methanol after 7-10 days of treatment, stained with 0.2% crystal violet solution (Sangon Biotech, #A100528) and photographed. Colonies comprising more than 50 cells were counted.

***Apoptosis Analysis***

Apoptosis measurements were performed to evaluate the effect of drugs on the apoptosis of different breast cancer cell groups. Cells were seeded in 6 cm plates and allowed to adhere. Drugs were added at 24 h for an additional 24 or 48 h. The cells were harvested by centrifugation at 800 rpm for 5 min, and the cell pellets were washed twice with precooled PBS (Basal Media, #B310). Next, the pellets were suspended in 100 μl of binding buffer and mixed with 10 μl of 7-AAD and 5 μl of Annexin V/PE (Yeason, #40310ES60) for 30 min in the dark. The mix was further diluted using binding buffer and apoptosis was determined by ﬂow cytometry analysis. The experiment was repeated three times under the same conditions.

***Immunofluorescence Assay***

The cells were washed three times with precooled PBS and ﬁxed in 4% paraformaldehyde (Yeason, #36314ES76) for 20 min. The cells were permeabilized with 0.5% Triton-X-100 for 20 min at 4 ℃. After rinsing with PBS three times, the cells were blocked for 1 h with 5% goat serum and incubated with anti-γ-H2A.X (1:500) antibody overnight at 4 ℃. The cells were rinsed with PBS three times and incubated with secondary antibody conjugated with Alexa 488 (Abcam, #150113) (1:500) at room temperature for 1 h. Next, the cells were washed with PBS three times, and sealed with DAPI-containing FluoroShield mounting medium (Abcam, #ab104139). Images were visualized using a Leica SP5 confocal microscope and analyzed. The γ-H2A.X-positive cell percent was calculated by (γ-H2A.X add-in cells/DAPI stained cells) × 100%. At least 500 cells were counted per well. Images were visualized using a Leica SP5 confocal microscope and analyzed.

***Homologous Recombination DNA Repair Assay***

A U2OS derivative clone stably expressing the HR reporter DR-GFP was used to assess the DNA repair ability^[1]^. U2OS-DR-GFP cells were seeded onto a 10 cm plate, followed by cotransfection with the I-SceI expression plasmid, MED23 WT and MED23 p.P394H expression plasmid. At 24 h after transfection, the medium was replaced with DMEM containing 10 μM triamcinolone acetonide (Selleck, #S1628) for another 48 h. The number of GFP-positive cells was measured by ﬂow cytometry using a Beckman FACSCanto II (BD).

**References**

[1] GUNN A, STARK J M. I-SceI-based assays to examine distinct repair outcomes of mammalian chromosomal double strand breaks [J]. Methods Mol Biol, 2012, 920(379-91.

**Table S1. Detailed information on the expression constructs (a), primers (b) and shRNA sequences (c).**

a)

| Plasmid | Source | Vector |
| --- | --- | --- |
| Flag-MED23 | Genomeditech (22486GR-P) | PGMLV-CMV-MCS-Flag-EF1 -T2A-Blasticidin |
| Flag-MED23 p.P394H | Genomeditech (22486GS-P) | PGMLV-CMV-MCS-Flag-EF1 -T2A-Blasticidin |
| shMED23 | Genechem (89454-89456) | PCMV-Puro-Amp |
| REL-blast | Genechem (GOSE 0240802) | PCMV-MCS-blasticidin-Amp |
| REL p.D268E-blast | Subcloned | PCMV-MCS-blasticidin-Amp |
| shREL | Subcloned | PLKO.1-TRC |

b)

|  | Primer | Sequence |
| --- | --- | --- |
| shREL#1 | Forward | CCGGGCAGGAATCAATCCATTCAATCTCGAGATTGAATGGATTGATTCCTGCTTTTTG |
|  | Backward | AATTCAAAAAGCAGGAATCAATCCATTCAATCTCGAGATTGAATGGATTGATTCCTGC |
| shREL#3 | Forward | CCGGCCACCTATATAGATGCAGCATCTCGAGATGCTGCATCTATATAGGTGGTTTTTG |
|  | Backward | AATTCAAAAACCACCTATATAGATGCAGCATCTCGAGATGCTGCATCTATATAGGTGG |
| REL p.D268E | Forward | CGGAGACCTTCTGAGCAGGAAGTTAGTG |
|  | Backward | CACTAACTTCCTGCTCAGAAGGTCTCCG |

c)

|  | Sequence |
| --- | --- |
| shREL#1 | GCAGGAATCAATCCATTCAAT |
| shREL#3 | CCACCTATATAGATGCAGCAT |
| shMED23#2 | CGCAGTTTACACGCTTCCTTA |
| shMED23#3 | CTGCAGCAGAGTCTAAGAAAT |

**Table S2. Vendors and working concentrations of the antibodies.**

| Antibody | Vendor | Cat# | Working Concentration |
| --- | --- | --- | --- |
| REL | Abcam | ab133251 | 1:10000(WB) |
| MED23 | Bethyl | A300-425A | 1:1000 (WB) |
| γ-H2A.X (Ser139) | Abcam | ab81299 | 1:5000 (WB) |
| p-ATM (Ser1981) | Abcam | ab81292 | 1:10000(WB) |
| p-CHK2 (Thr68) | CST | 2661S | 1:1000 (WB) |
| p-ATR (Ser428) | Abcam | ab178407 | 1:1000 (WB) |
| DNA-PKcs (Y393) | Abcam | ab32566 | 1:1000 (WB) |
| Vinculin | Sigma | V9131 | 1:5000 (WB) |
| β-actin | CST | 3700 | 1:5000 (WB) |
| γ-H2A.X | Abcam | ab22551 | 1:500 (IF) |
| Mouse IgG | Abcam | ab150113 | 1:200 (IF) |
| Mouse IgG, HRP-linked | CST | 7076 | 1:5000 (WB) |
| Rabbit IgG, HRP-linked | CST | 7074 | 1:5000 (WB) |

**Figure S1.** Principal component analyses of radiomic features from 3 types of MRI machines.


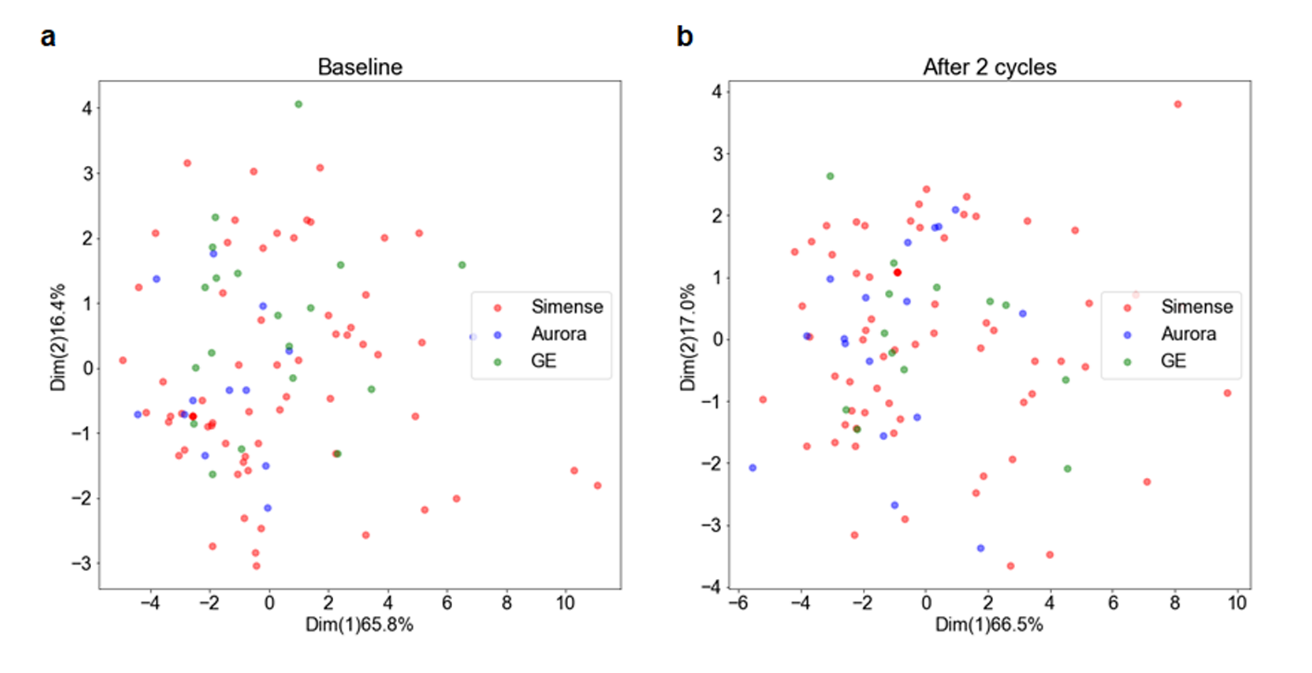


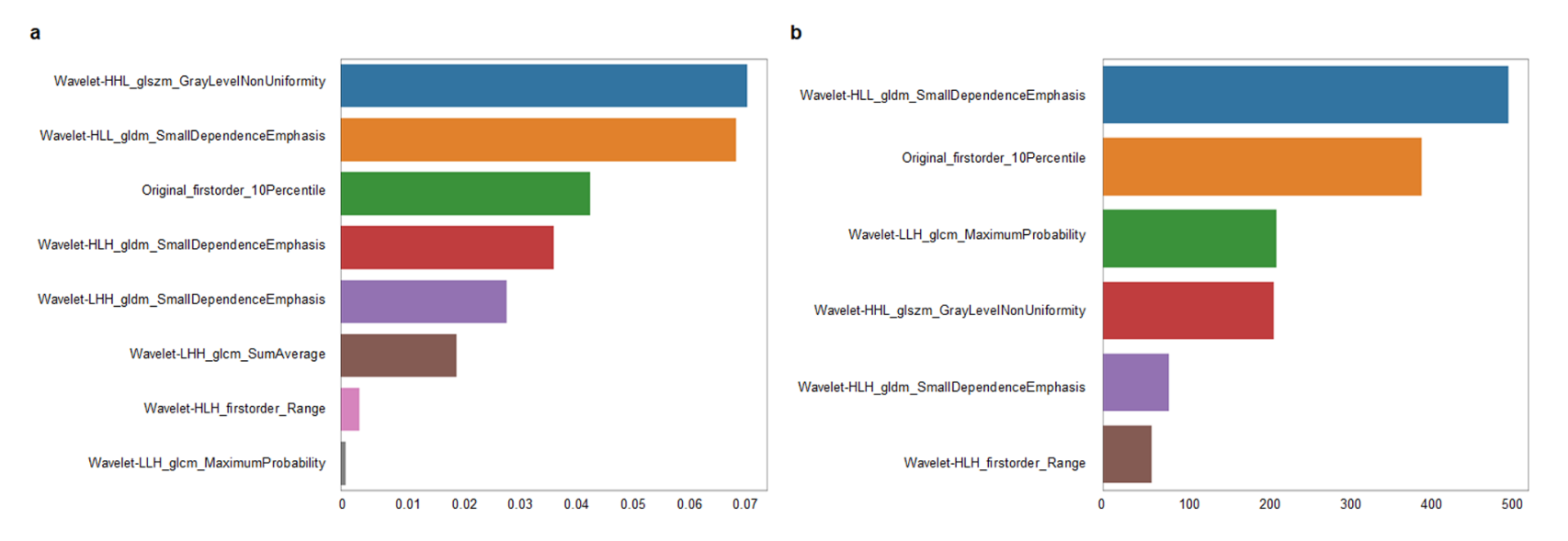
**Figure S2.** Details of radiomic feature extraction using LASSO (a) and XGBoost (b) after 2 cycles.

Two feature selection steps were applied to the extracted radiomic features with the least absolute shrinkage and selection operator (LASSO) and XGBoost.

(a) The LASSO model is a linear combination of the selected features weighted by their respective coefficients. The x-axis denotes LASSO coefficients. Features with nonzero coefficients denote greater contributions to the model and are selected.

(b) Feature importance evaluates how valuable each feature was in the construction of the gradient boosted decision trees within the XGBoost model and is calculated by information gain. The x-axis measures the information gain.

**Figure S3.** ROC curves of the radiomic model at baseline (green) and after 2 cycles (yellow).

1. Training set
2. Validation set


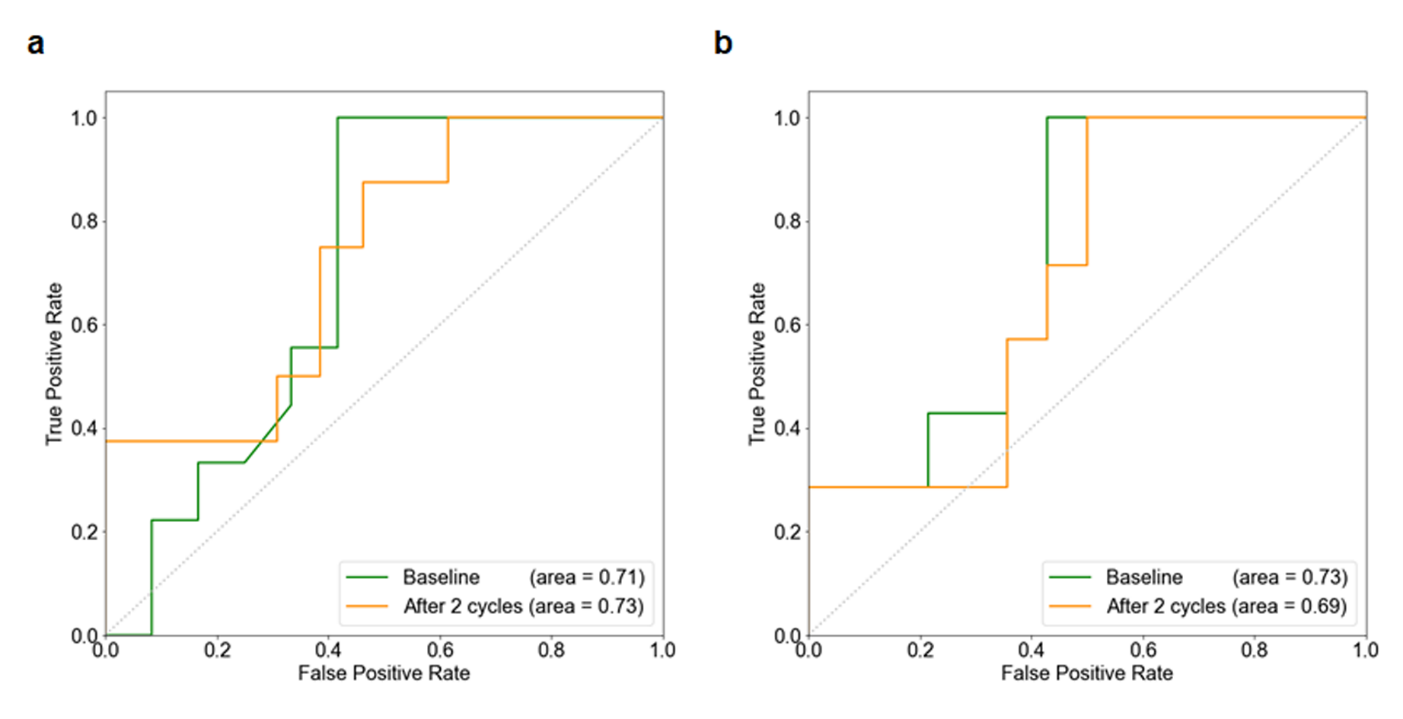


**Figure S4.** Mutation profile oncoplot of the study population.


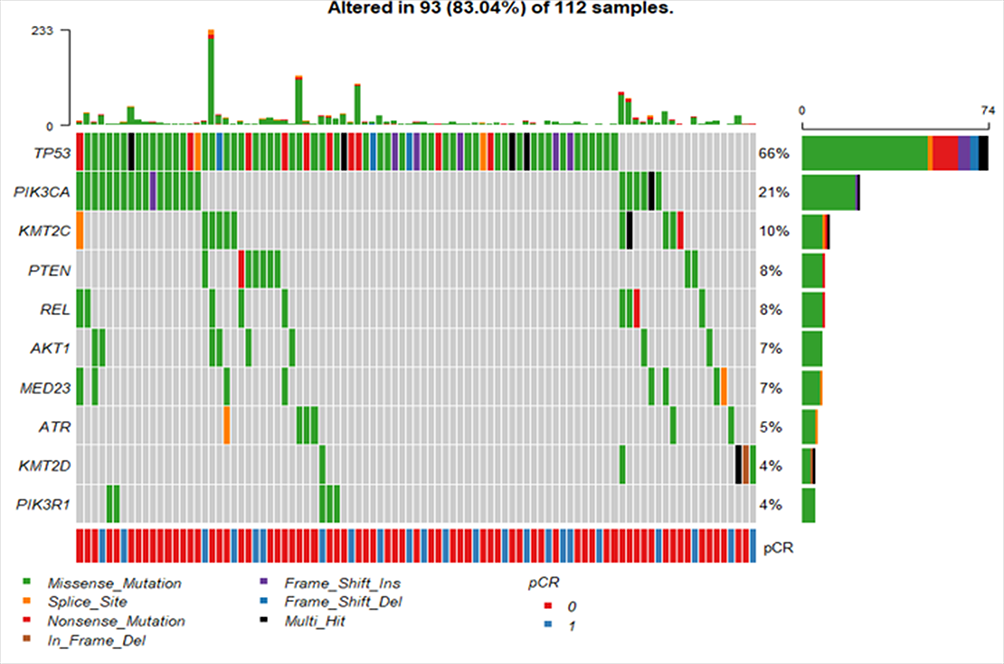


**Figure S5.** Selection of genomic features using XGBoost.


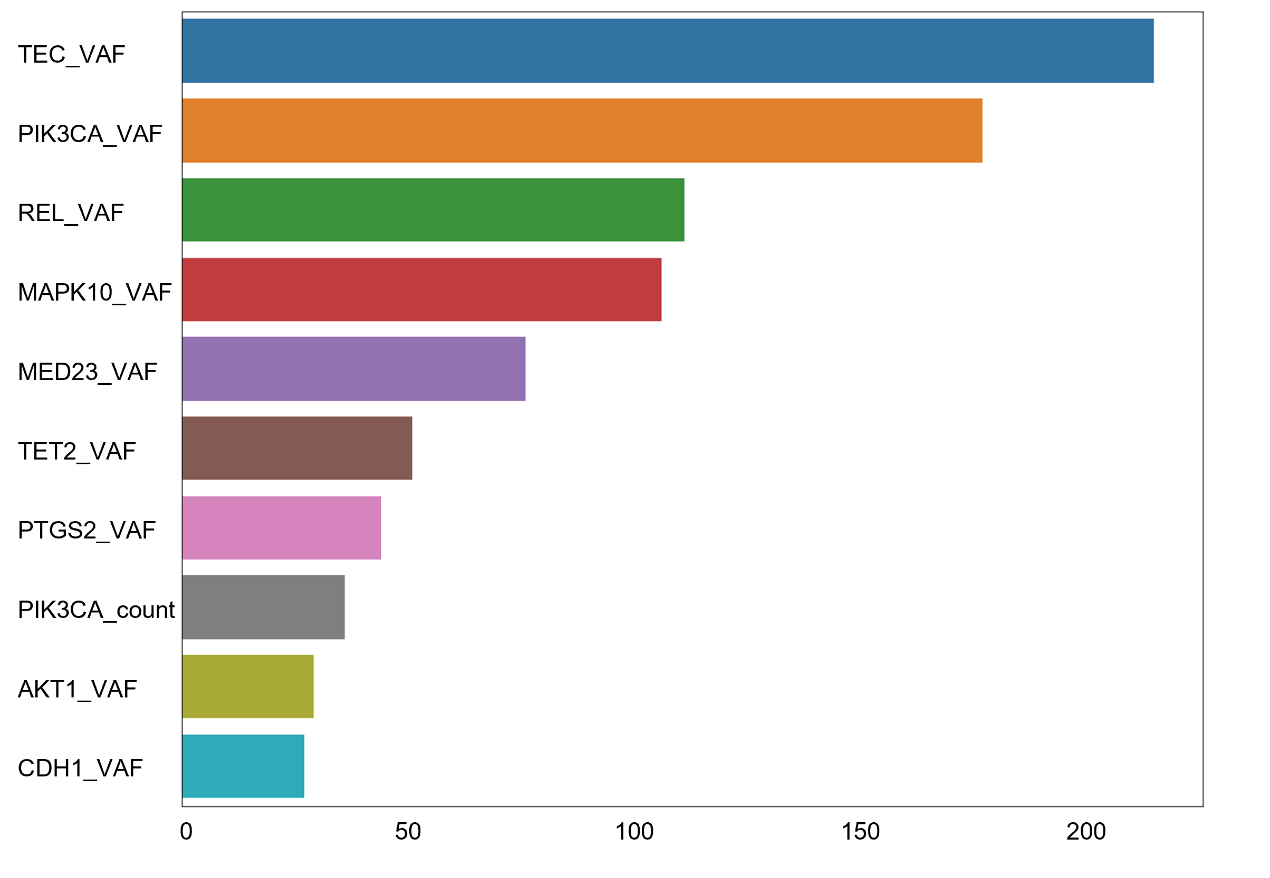


For genomic feature selection, variables including mutation status (positive or negative), detected mutation counts and VAF of each gene were input. After standardization, feature importance evaluates how valuable each feature was in the construction of the gradient boosted decision trees within the XGBoost model and is calculated by information gain. The x-axis measures the information gain. Features with the top 10 information gain rankings are presented. The selection criterion was defined as an information gain over 75. Therefore, 5 VAF features were selected.

**Figure S6**. IC_50_ and colony formation assay with epirubicin and paclitaxel treatment in stable cells expressing wild-type REL and D268E mutation.

(A) REL mutations discovered in this cohort. REL p.D268E was identiﬁed as a recurrent spot.

(B) Stable shREL SUM-159 and MDA-MB-231 cells were further transfected with wild-type REL or p.D268E mutation, respectively, and subjected to immunoblotting.

(C-F) SUM-159 and MDA-MB-231 cells stably expressing wild-type REL and the D268E mutation were treated with increasing doses of epirubicin and subjected to colony formation survival assays. Representative images of surviving colonies are shown in C and D, and the corresponding quantitative results are shown in E and F.

(G-H) SUM-159 and MDA-MB-231 cells stably expressing wild-type REL and the D268E mutation were treated with increasing doses of epirubicin and subjected to IC_50_ assays.

**p*< 0.05, ***p*< 0.01, ****p*< 0.001

(I-L) SUM-159 and MDA-MB-231 cells stably expressing wild-type REL and D268E mutation were treated with increasing doses of paclitaxel and subjected to colony formation survival assays. Representative images of surviving colonies are shown in I and J, and the corresponding quantitative results are shown in K and L.

(M-N) SUM-159 and MDA-MB-231 cells stably expressing wild-type REL and the D268E mutation were treated with increasing doses of paclitaxel and subjected to IC_50_ assays.

**p*< 0.05, ***p*< 0.01, ****p*< 0.001

**
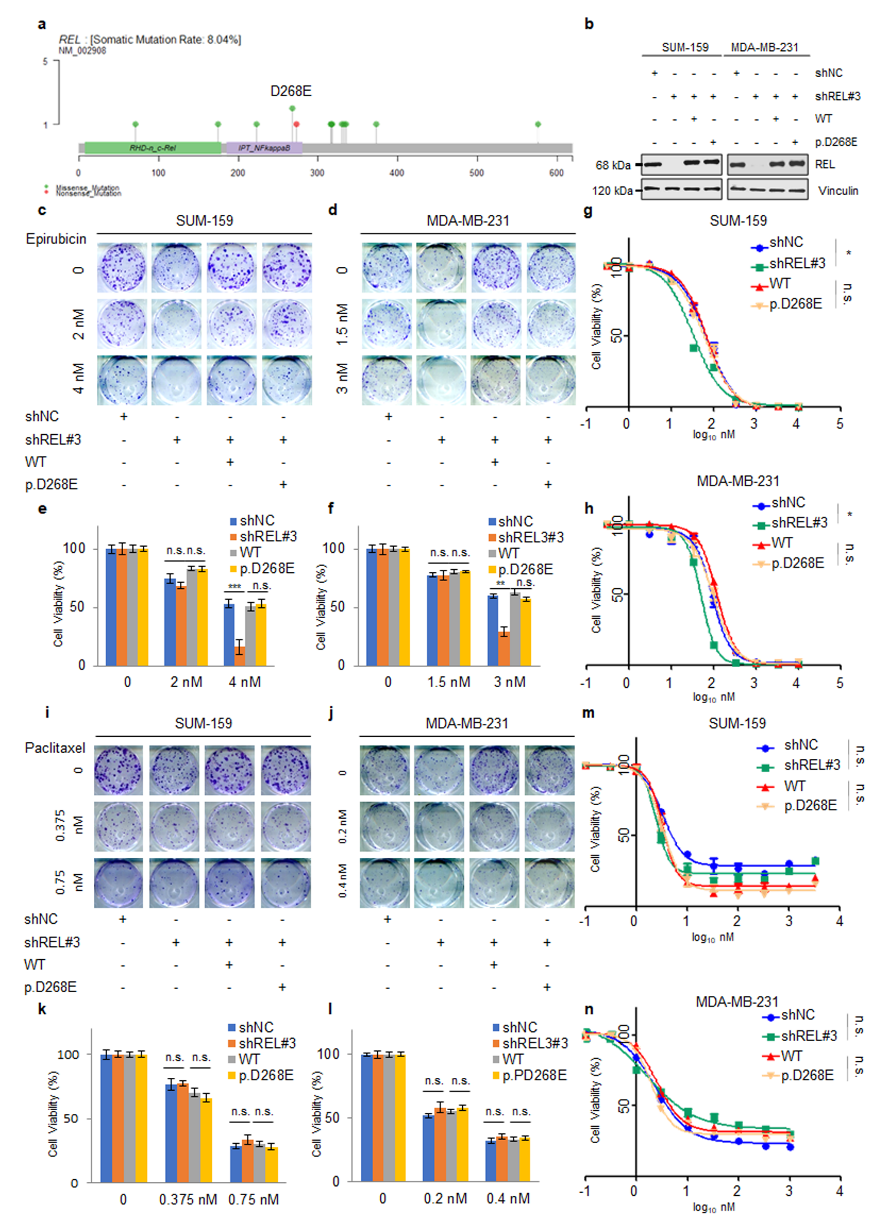
**

**Figure S7.** Construction of MED23 (a) and REL (b) knockdown cell lines.

(a) SUM-159 and BT-549 cells were transfected with shMED23 and shNC. After selection by puromycin, the cells were collected and subjected to immunoblotting.

**
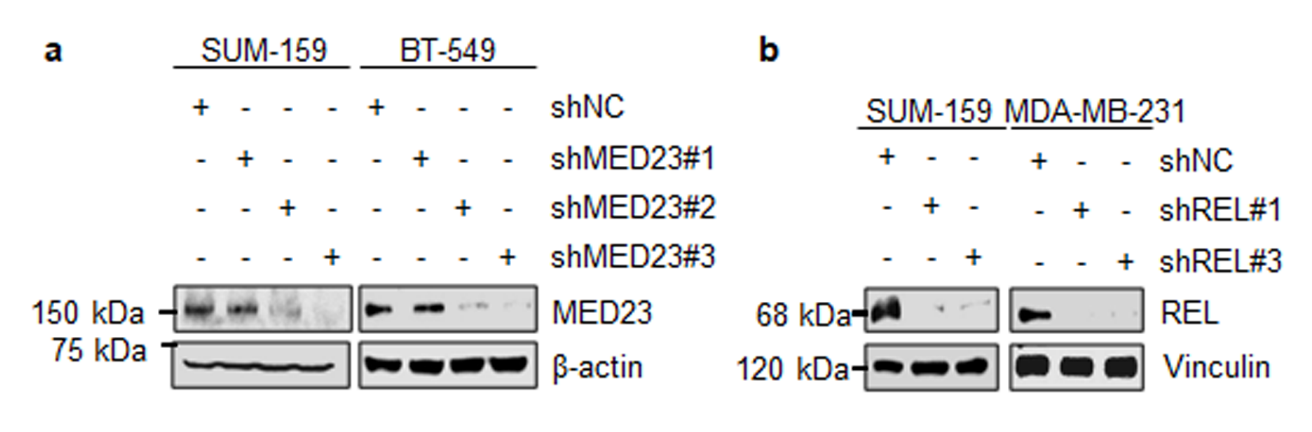
**(b) SUM-159 and MDA-MB-231 cells were transfected with shREL and shNC. After 48 h of transfection, the cells were treated with puromycin for 7-10 days at a concentration of 5 µg/ml and then subjected to immunoblotting.

**Figure S8**. IC_50_ and colony formation assay with paclitaxel treatment in stable cells expressing wild-type MED23 and P394H mutation.

(A-D) MED23 was knocked down via shRNA. SUM-159 and BT-549 cells stably expressing wild-type MED23 and P394H mutation were treated with increasing doses of paclitaxel and subjected to colony formation survival assays. Representative images of surviving colonies are shown in A and C, and the corresponding quantitative results are shown in B and D.

(E-F) SUM-159 and BT-549 cells stably expressing shNC, shMED23, wild-type MED23 and P394H mutation were treated with increasing doses of paclitaxel and subjected to IC_50_ assays.

**p*< 0.05, ***p*< 0.01, ****p*< 0.001

**
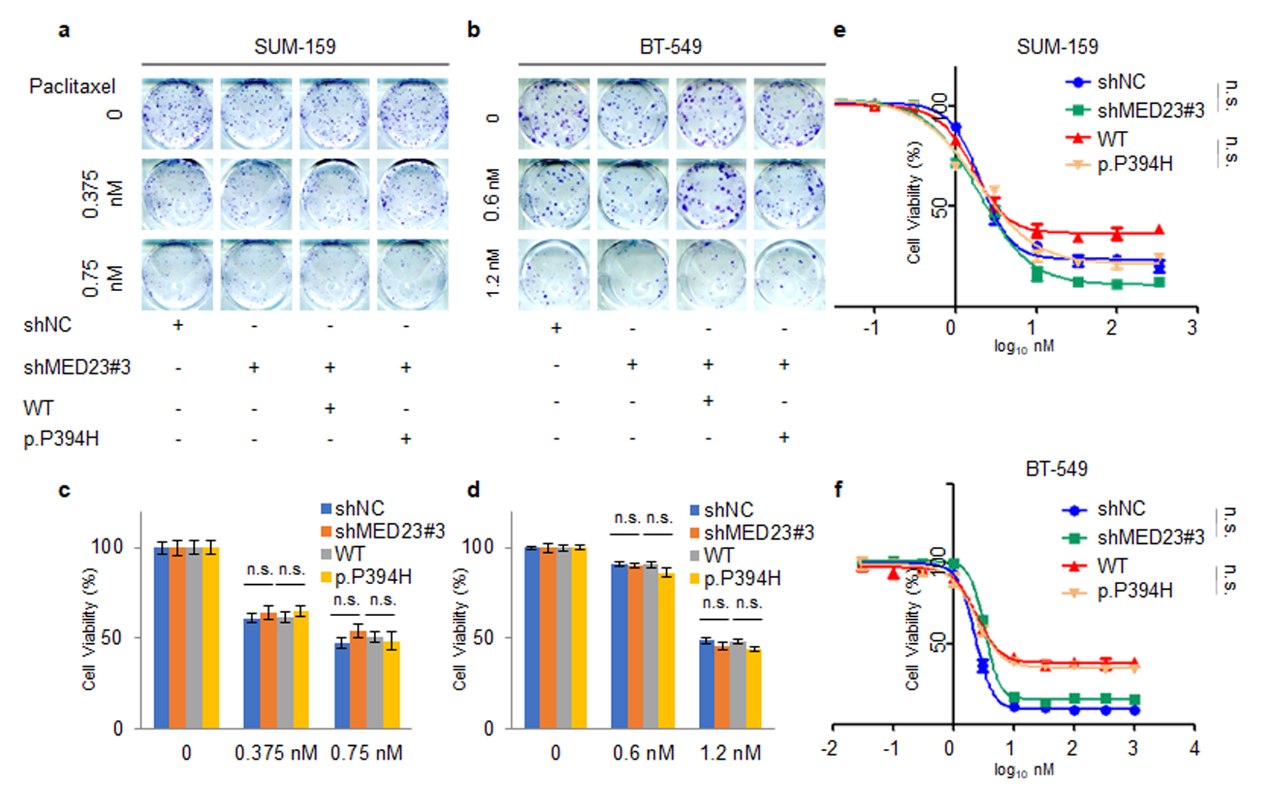
**

**Figure S9**. Apoptosis of SUM-159 and BT-549 cells stably expressing wild-type MED23 and p.P394H mutation after epirubicin treatment.

The upper right quadrant represents dead/late apoptotic cells, whereas the lower right quadrant represents early apoptotic cells. After treatment with epirubicin (10 nM, 24 h), the cells were stained with Annexin V/PE and 7AAD, and observed by flow cytometry.

**p*< 0.05, ***p*< 0.01, ****p*< 0.001

**
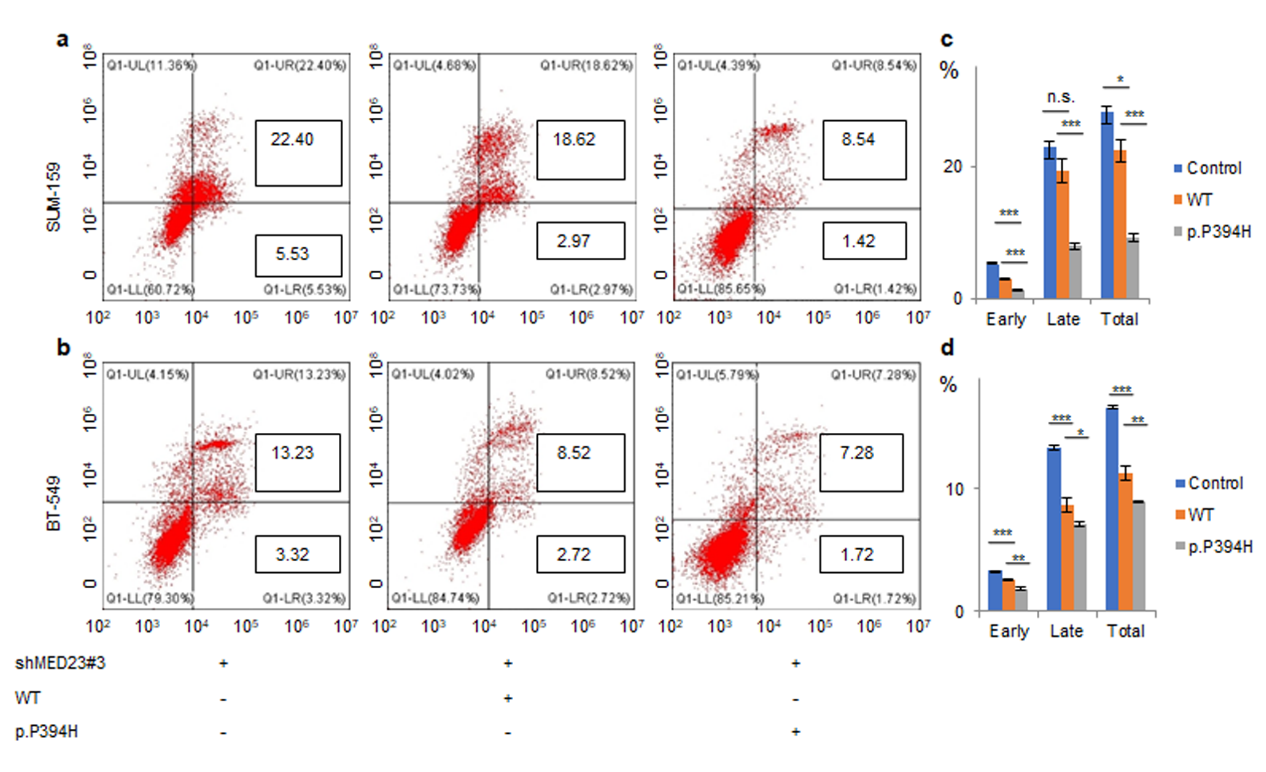
**

**Figure S10**. MED23 p.P394H promoted homologous recombination repair.

(a) U2OS cells expressing wild-type MED23 and the P394H mutation were transfected with I-SceI. After 24 h, the cells were treated with 10 μM triamcinolone acetonide for another 48 h. The HR reporter assay was determined by ﬂow cytometry.

(b) The working model of the HR reporter system is presented. I-SceI endonuclease was introduced into GFP/U2OS cells.

(c) Quantification of GFP-positive cells. ***p*< 0.01

**
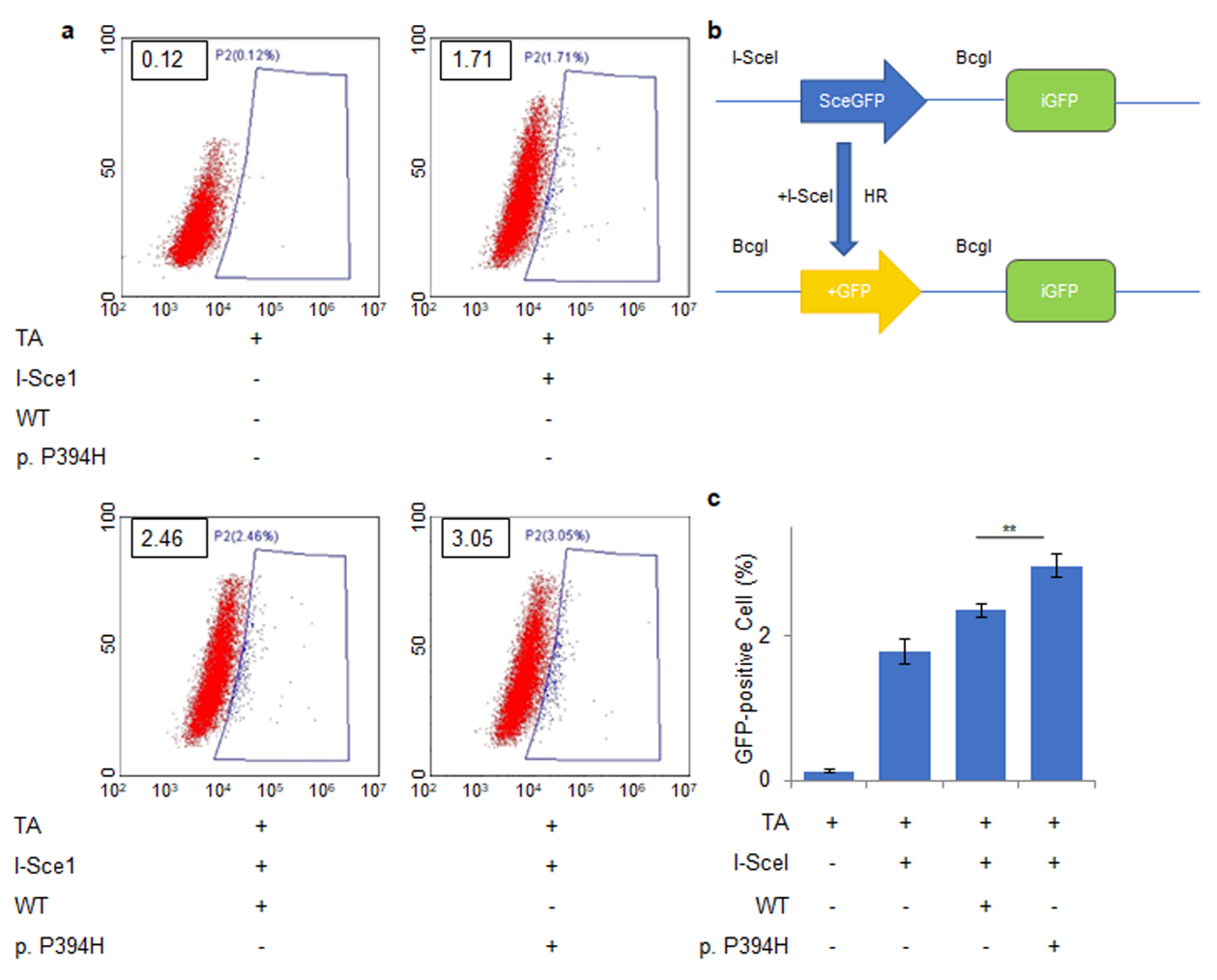
**

**Original images from Western Blotting.**

**Figure S4b**

MED23


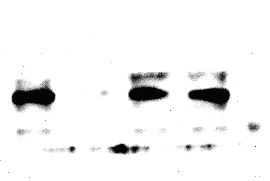

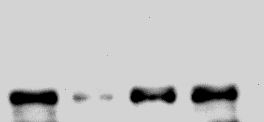


β-actin


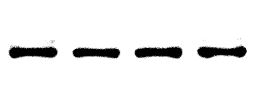

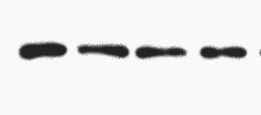


**Figure S5a**

MED23


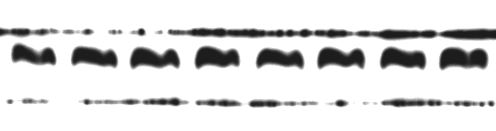

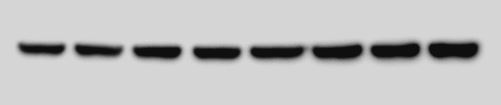


γ-H2A.X


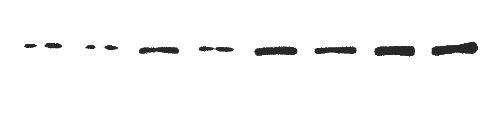


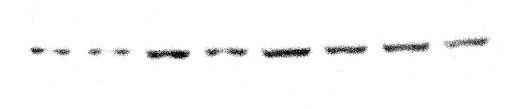


p-ATM (wk)


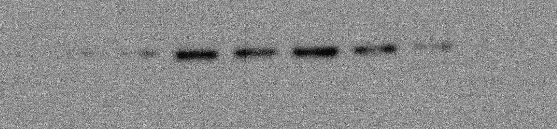


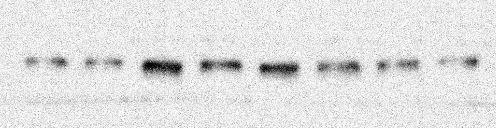


p-ATM (str)


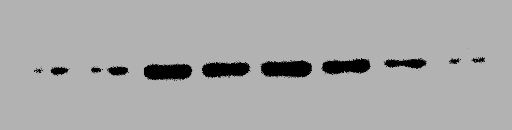


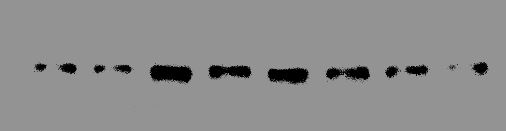


p-CHK2


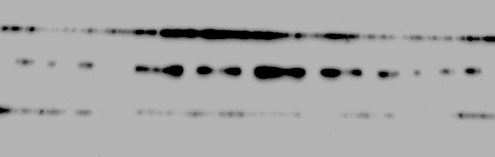


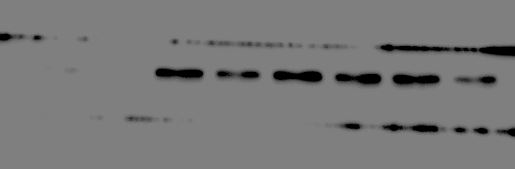


p-ATR


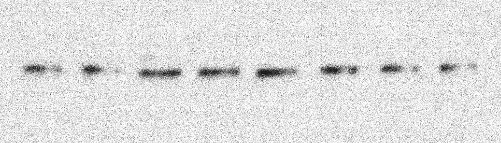


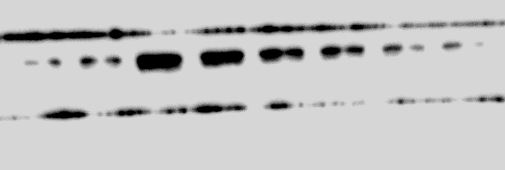


DNA-PKcs


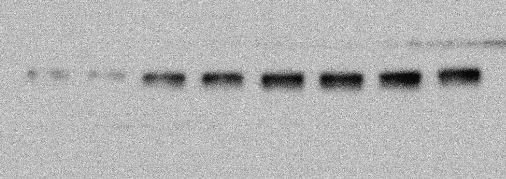


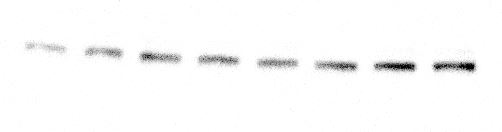


β-actin

**
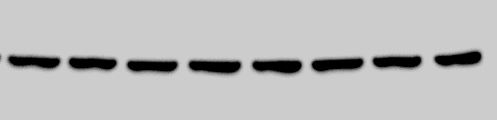
**

**
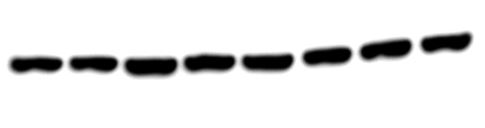
**

**Fig. S6b**

REL


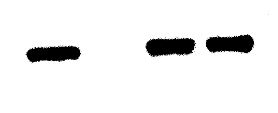

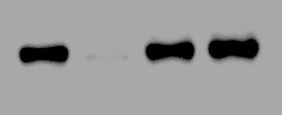


Vinculin


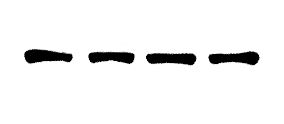

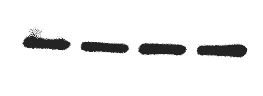


**Fig. S7**

MED23


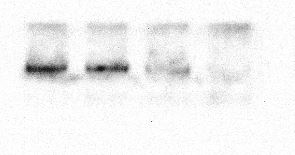

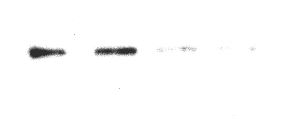


β-actin


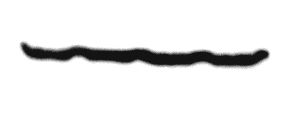

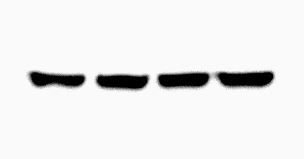


REL


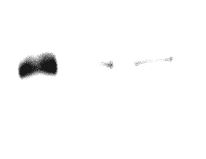

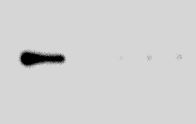


Vinculin

**
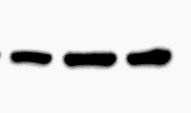

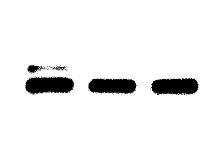
**
